# Impact of SO_x_, NO_x_ and NH_3_ emission reductions on PM_2.5_ concentrations across Europe: Hints for future measure development

**DOI:** 10.1016/j.envint.2021.106699

**Published:** 2021-11

**Authors:** A. Clappier, P. Thunis, M. Beekmann, J.P. Putaud, A. de Meij

**Affiliations:** a Université de Strasbourg, Laboratoire Image Ville Environnement, Strasbourg, France; b European Commission, Joint Research Centre, Ispra, Italy; c Université de Paris and Université Paris-Est Créteil, CNRS, LISA, F-75013 Paris, France; d MetClim Varese, Italy

**Keywords:** European air quality planning, PM formation, Chemical regimes, Non-linearity

## Abstract

Given the remaining air quality issues in many European regions, smart air quality strategies are necessary to reduce the burden of poor air quality. While designing effective strategies for non-reactive primary pollutants is straightforward, this is not the case for secondary pollutants for which the relationship between emission changes and the resulting concentration changes can be nonlinear. Under such conditions, strategies targeting the largest emitting sources might not be the most effective. In this work, we provide elements to better understand the role of the main emission precursors (${SO}_{2}$, ${NO}_{x}$, ${NH}_{3}$) on the formation of secondary inorganic aerosols. By quantifying the ${PM}_{2.5}$ sensitivity to emission reductions for each of these three precursors, we define and quantify the intensity of ${PM}_{2.5}$ formation chemical regimes across Europe. We find that for emission reductions limited to 25%, the relation between emission and PM concentration changes remain mostly linear, with the exception of the Po Valley where non-linearities reach more than 30% in winter. When emission reductions increase to 50%, non-linearity reaches more than 60% in the Po Valley but stay below 30% in the rest of Europe. In terms of implications on abatement strategies, our findings can be summarized in the following key messages: (1) reducing ${SO}_{2}$ emissions where abundant is always efficient (e.g. eastern Europe and Balkans); (2) reducing ${NH}_{3}$ emissions is more efficient where it is less abundant (e.g. the Po basin) than where it is abundant, given the limiting role of ${NH}_{3}$ in the PM formation; (3) reducing ${NO}_{x}$ emissions where ${NO}_{x}$ are abundant can be counter-productive with potential increases of PM due to the increased oxidant capacity of the atmosphere (e.g. Po valley); (4) because regions with both ${NH}_{3}$ and ${NO}_{x}$ sensitive chemical regimes are mixed within countries, both need to be reduced together, as pollution reduction policies need at least to be defined at a country level; (6) while for ${NH}_{3}$ the focus is clearly on wintertime, it is the whole year for ${NO}_{x}$. The simulations proposed in this work could be used as benchmark for other models as they constitute the type of scenarios required to support air quality strategies. In addition, the straight and systematic emission reductions imposed for the scenarios in this work are well suited for a better understanding of the behavior of the model, in terms of responses to emission reductions.

## Highlights

• For moderate reductions, emission and PM concentration changes are linearly linked.
• Reducing ${SO}_{2}$ emissions where abundant is always efficient.
• Reducing ${NH}_{3}$ emissions is more efficient where it is less abundant.
• Reducing ${NO}_{x}$ emissions where ${NO}_{x}$ are abundant can be counter-productive.
• Both ${NO}_{x}$ and ${NH}_{3}$ regimes occur in some regions, calling for combined reductions

## 1 Introduction

Although air quality steadily improves in Europe, several regions still experience levels that exceed both the values recommended by the WHO and the limit values listed in the EU air quality directives. This is the case in particular for fine particulate matter mass concentrations (${PM}_{2.5}$) for which both the EU limit values (yearly average of 25 μg/m3) and WHO recommended guidelines (yearly average of 10 μg/m3) are yet exceeded in many urban areas, especially in Eastern Europe, Northern Italy and in the Benelux area (Guerreiro et al., 2018). This is critical as long-term exposure to ${PM}_{2.5}$ is associated with *i.a.* an increased risk of cardiopulmonary mortality, responsible for the majority of the 390 000 premature deaths currently reported per year in the EU28 (EEA, 2020).

Given the remaining air quality issues in many European regions, smart air quality strategies (i.e. strategies that are effective in reducing concentrations below exceedance thresholds) shall be implemented to reduce the burden of poor air quality. Air quality strategies are applied at all spatial scales, from European scale (e.g. the National Emission Ceiling Directives, Gothenburg protocol) to the very local scale (implementation of low emission zones in many European cities). To be effective, emission abatement measures must target the right emission sources. This question is easily addressed when primary pollutants only are involved, as these pollutants respond linearly to emission changes, i.e. concentration reductions are proportional to the imposed emission abatement. In these conditions, strategies targeting the largest emitting sources will be the most effective. The challenge is however more complex when secondary pollutants are involved and the relationship between emission changes and resulting concentration changes is nonlinear. In these situations, targeting the largest emission sources may not be effective, and even be counter-productive (i.e. leading to an increase of the concentration levels). The formation of ozone (O_3_), which is totally of secondary origin, represents a perfect example of such situations and a large amount of research have helped to design abatement strategies for this pollutant by defining different $O_{3}$ formation chemical regimes. For pollutants that form from various precursors (as $O_{3}$ forms from volatile organic compounds VOC and nitrogen oxides ${NO}_{x}$), chemical regimes are defined according to the precursor that controls the targeted pollutant levels (VOC or ${NO}_{x}$-limited regimes for $O_{3}$). For example, Sillman (1999) reviews several decades of works on chemical regimes starting from the Los Angeles Basin and extending to polluted rural areas in California (USA). Beekmann and Vautard (2010) provide a comprehensive study on $O_{3}$ formation chemical regimes over Europe. They note that during summer time, VOC limited regimes are present especially over urbanized areas (in particular north-western Europe and the Po valley in Italy) while ${NO}_{x}$ (nitrogen oxides) sensitive chemical regimes occur over southern Europe, especially the Mediterranean region.

In the case of ${PM}_{2.5}$, both primary and secondary particulate species contribute to the overall concentration of ${PM}_{2.5}$. Primary particulates are directly emitted in the air as particles. Secondary particulates are formed by chemical or photochemical reactions from gaseous or particulate precursors. The secondary ${PM}_{2.5}$ fraction is generally the largest (Aksoyoglu et al., 2017). Secondary inorganic aerosols (SIA) result from chemical reactions that involve gaseous precursors: ${SO}_{2}$ (sulfur oxides), ${NO}_{x}$ and ${NH}_{3}$. The responses of PM concentration to ${SO}_{2}$/${NO}_{x}$/${NH}_{3}$ emission reductions show non-linear or at least non-proportional behaviours (e.g. Seinfeld and Pandis, 2006). For example, a strong decrease of ${SO}_{2}$ emissions will lead to a lesser sulfate formation, freeing ${NH}_{3}$ that can form ammonium nitrate. This process, which was both observed and modelled, has been used to explain the limited nitrate decrease resulting from simultaneous ${NO}_{x}$ and ${SO}_{2}$ emission reductions both in the US (Shah et al., 2018), Europe (Ciarelli et al., 2019) and lately China (Lachatre et al., 2019). Non-linear behaviours often result from chemical regimes in which a formation process is limited by the less abundant species. Although other processes may take place, the formation of ammonium nitrate is for example mostly controlled by the less abundant species between ammonia and nitric acid. It is interesting to note that in these cases, strategies should *a priori* target the limiting species, although this species is already less abundant. In practice, not only ${PM}_{2.5}$ but also the concentrations of some of its precursors are regulated (e.g. ${NO}_{x}$, ${SO}_{2}$…) so that most emission sources are targeted. This is however not the case for all precursors (e.g. ${NH}_{3}$).

The aim of this work is to identify the chemical regimes in which secondary inorganic PM is formed over Europe, and to analyse how the knowledge of these chemical regimes can help in designing efficient PM abatement strategies. In particular we analyse the robustness of these regimes in terms of temporal and spatial variabilities, and in terms of dependence on the level of emission reductions that is imposed.

The structure is organised as follows. We first describe the mathematical approach and the model simulations used to determine the sensitivity and linearity of ${PM}_{2.5}$concentration responses to the abatement of gaseous precursor emissions (Section 2). Section 3 first details the chemical regimes themselves and highlight non-linear responses in a second part. Section 4 discusses the implications of our findings in terms of policy making. Conclusions are drawn in Section 5.

## 2 Methodology

### 2.1 Mathematical formulation

To express in a harmonized and comparable way concentration changes resulting from emission reductions from different factors and/or different intensity, we use here the concept of potential impact. Potential impacts (*P*) are defined (Thunis et al., 2020) as the ratio between the concentration change and the emission reduction intensity:

(1) $$P_{NH3}^{\alpha }=\frac{\Delta C_{NH3}^{\alpha }}{\alpha };P_{\mathrm{NOx}}^{\alpha }=\frac{\Delta C_{\mathrm{NOx}}^{\alpha }}{\alpha };P_{SO2}^{\alpha }=\frac{\Delta C_{SO2}^{\alpha }}{\alpha }$$

where $\Delta C_{NH3}^{\alpha }$, $\Delta C_{\mathrm{NOx}}^{\alpha }$ and $\Delta C_{SO2}^{\alpha }$ are the PM concentration change resulting from a reduction of the ${NH}_{3}$, ${NO}_{x}$ and ${SO}_{2}$ emissions, respectively. α is the emission reduction intensity that varies from 0 (no reduction) to 1. When α=1 the potential impact is equal to the concentration change due to a full emission reduction.

In Annex A we state that the overall potential impact computed for a simultaneous reduction of all precursors ($P_{\mathrm{all}}^{\alpha }=P_{NH3}^{\alpha }+P_{\mathrm{NOx}}^{\alpha }+P_{SO2}^{\alpha }$) can be split into the following components:

(2) $$P_{\mathrm{all}}^{\alpha }=P_{\mathrm{all}}^{\mathrm{lin}}+{\hat{P}}_{\mathrm{tot}}^{\alpha }$$

where $P_{\mathrm{all}}^{\mathrm{lin}}$ and ${\hat{P}}_{\mathrm{tot}}^{\alpha }$ are the linear component and the non-linear deviation term, respectively.

For practical reasons, in the context of this work the relationship between emissions and concentrations is linear when the two following conditions are fulfilled:

**Consistency**: each precursor’s potential impact remains constant over the entire range of emission reductions (i.e. ∀α).
**Additivity**: the overall potential impact (due to the simultaneous reduction of all the precursors) is equal to the sum of the single precursor’s potential impacts.

In Annex A we explain that the potential impacts computed for a “low enough” percentage emission reduction (i.e. α=0.1) behave quasi linearly.

(3) $$P_{\mathrm{all}}^{\mathrm{lin}}\approx P_{NH3}^{0.1}+P_{\mathrm{NOx}}^{0.1}+P_{SO2}^{0.1}$$

The non-linear deviation terms can be split into the following components (see Annex A for details):

(4) $${\hat{P}}_{\mathrm{tot}}^{\alpha }={\hat{P}}_{NH3}^{\alpha }+{\hat{P}}_{\mathrm{NOx}}^{\alpha }+{\hat{P}}_{SO2}^{\alpha }+{\hat{P}}_{NH3-NOx}^{\alpha }+{\hat{P}}_{NH3-SO2}^{\alpha }+{\hat{P}}_{\mathrm{NOx}-SO2}^{\alpha }+{\hat{P}}_{NH3-NOx-SO2}^{\alpha }$$

where ${\hat{P}}_{NH3}^{\alpha };{\hat{P}}_{\mathrm{NOx}}^{\alpha };{\hat{P}}_{SO2}^{\alpha }$ represent the single precursor’s non-linear deviation terms, computed as the difference of potential impacts between a given level α and the linear level (0.1):

(5) $${\hat{P}}_{NH3}^{\alpha }={P_{NH3}^{\alpha }-P}_{NH3}^{0.1};{\hat{P}}_{\mathrm{NOx}}^{\alpha }={P_{\mathrm{NOx}}^{\alpha }-P}_{\mathrm{NOx}}^{0.1};{\hat{P}}_{SO2}^{\alpha }={P_{SO2}^{\alpha }-P}_{SO2}^{0.1}$$

Some terms cannot be associated to the reduction of a single precursor: ${\hat{P}}_{NH3-NOx}^{\alpha };{\hat{P}}_{NH3-SO2}^{\alpha };{\hat{P}}_{\mathrm{NOx}-SO2}^{\alpha }$ are the non-linear deviation terms linked to the simultaneous reduction of two precursors:

(6) $${\hat{P}}_{NH3-NOx}^{\alpha }=P_{NH3-NOx}^{\alpha }-P_{NH3}^{\alpha }-P_{\mathrm{NOx}}^{\alpha }$$

(7) $${\hat{P}}_{NH3-SO2}^{\alpha }=P_{NH3-SO2}^{\alpha }-P_{NH3}^{\alpha }-P_{SO2}^{\alpha }$$

(8) $${\hat{P}}_{\mathrm{NOx}-SO2}^{\alpha }=P_{\mathrm{NOx}-SO2}^{\alpha }-P_{\mathrm{NOx}}^{\alpha }-P_{SO2}^{\alpha }$$

${\hat{P}}_{NH3-NOx-SO2}^{\alpha }$ is the non-linear deviation term that can only be associated to the effect of the reduction of all three precursors.

(9) $${\hat{P}}_{NH3-NOx-SO2}^{\alpha }=P_{\mathrm{all}}^{\alpha }-P_{NH3}^{\alpha }-P_{\mathrm{NOx}}^{\alpha }-P_{SO2}^{\alpha }-P_{NH3-NOx}^{\alpha }-P_{NH3-SO2}^{\alpha }-P_{\mathrm{NOx}-SO2}^{\alpha }$$

In this work, we calculate these various components with a chemical transport model and systematically analyze their spatial and temporal variations.

### 2.2 Model description and set up

In this study we use the off-line regional transport chemistry EMEP model version rv_33 an (Simpson et al., 2012; https://github.com/metno/emep-ctm), to analyse the relationship between air pollutant emissions and concentrations over Europe. The domain stretches from −15.05° W to 36.95° E longitude and 30.05° N to 71.45° N latitude with a horizontal resolution of 0.1° × 0.1° and 20 vertical levels, with the first level between 0 and ~ 7 m. The EMEP model uses meteorological initial conditions and lateral boundary conditions from the European Centre for Medium Range Weather Forecasting (ECMWF-IFS) for the meteorological year 2015. The temporal resolution of the meteorological input data is daily, with 3-hour timestep. The meteorological fields for EMEP are retrieved on 0.1° × 0.1° longitude latitude coordinate projection. Vertically, the fields on 60 eta (η) levels from the IFS model are interpolated on to the 20 EMEP sigma (σ) levels. The MARS equilibrium module is used calculate the partitioning between gas and fine-mode aerosol phase in the system of ${SO}_{4}^{2-}-{HNO}_{3}-{NO}_{3}^{-}-{NH}_{3}-{NH}_{4}^{+}$ (Binkowski and Shankar, 1995). More information on the gas and aerosol portioning is given in Simpson et al. (2012), Section 7.6. Detailed information on the meteorological driver, land cover, model physics and chemistry are described in Simpson et al. (2012) and in the EMEP Status Report 2017 (https://emep.int/publ/reports/2017/EMEP_Status_Report_1_2017.pdf).

In this study we use the aerosol and aerosol gaseous precursor emissions from the Copernicus Atmospheric Monitoring Service (CAMS) REG-v2.2.1 (0.1° × 0.05° lon × lat in Gridding Nomenclature For Reporting [GNFR] categories), for the year 2015. The CAMS inventory contains annual emissions for ${SO}_{2}$, ${NO}_{x}$ (as ${NO}_{2}$), ${NH}_{3}$, ${CH}_{4}$, non-methane volatile organic compounds (NMVOC), CO, ${PM}_{2.5}$ and ${PM}_{10}$ for each GNFR source sector. More information on the data collection and spatial allocation of the emissions are provided in Granier et al. (2019). The emissions are temporally distributed as described in Simpson et al. (2012). Emission data can be downloaded from https://eccad.aeris-data.fr.

Note that the scope of this work is not to evaluate the model performance in terms of modeled versus measured gas and aerosol concentrations. This evaluation work is detailed in other publications. The model performance in aerosol calculations has been discussed in Thunis et al., 2021a, Thunis et al., 2021b, and in the EMEP Status Report 2017. In Pisoni et al. (2021) calculated PM2.5 concentrations are evaluated by comparing with observations of the EIONET network (https://www.eea.europa.eu/data-and-maps/data/aqereporting-8). They showed that in general, calculated PM2.5 values are in better agreement with the observations than simulations with the EMEP emission inventory and CAMS-REG-AP emission inventory, especially in Eastern Europe. Regarding SIA, the model tends to overestimate in general the secondary inorganic aerosols (as described in the EMEP Status Report 2017).

### 2.3 Modelled scenarios

The EMEP model is used to simulate the scenarios needed to computed the various components of the potential impacts for α=0.25 and α=0.5:

• The base case (simulation of the current situation),
• scenarios reducing 10% (α=0.1) of the emissions, one for each single precursor
• 7 scenarios reducing 25% (α=0.25) of the emissions, 3 for the reduction of each precursor, 3 for the reduction of two precursors (${NO}_{x}$ & ${SO}_{2}$ ; ${NO}_{x}$ & ${NH}_{3}$ ; ${NH}_{3}$ & ${SO}_{2}$) and 1 for the reduction of all precursors (${NO}_{x}$ & ${SO}_{2}$ & ${NH}_{3}$).
• Similarly to the 25% reduction level, 7 additional scenarios reducing 50% of emissions to compute the potential impacts for α=0.5

To highlight the seasonal dependency of the impact of emission reductions on aerosol concentrations, we focus our analysis on three main periods in 2015, i.e. the summer period (May till September), the winter period (November till February), and a transition period (March, April and October).

## 3 Results

In this Section, the spatial and temporal variations of the chemical regimes and associated non-linearities are analyzed through the potential impact components expressed in Section 2.1. The analysis is made in two steps that correspond to two terms of Eq. (2): the linear component and the non-linear deviation term. We start by providing information about the base case concentration fields.

### 3.1 Base case

**Fig. 1:**
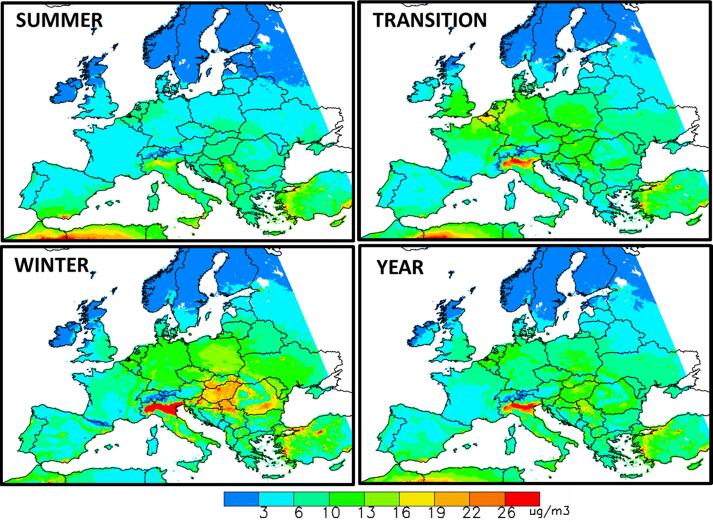
Maps of ${PM}_{2.5}$concentrations, Top-left: Summer (May to September), bottom-left Winter (November to February), top-right: Transition months (Mars, April, October) and bottom-right: Year average.

In general, the highest ${PM}_{2.5}$ occur during winter in the Po Basin (northern Italy), the Balkans and Turkey, with mean grid cell concentrations up to >26 µg/m^3^ (Fig. 1). This corroborates the results of previous studies, which have shown that this region is one of the most polluted in Europe because it is highly industrialized and densely populated and because of its peculiar orography and meteorological conditions (De Meij et al., 2009a, De Meij et al., 2009b, Larsen et al., 2012, Thunis et al., 2013 and 2020; Bigi and Ghermandi, 2016, Arvani et al., 2014, Putaud et al., 2014, Diémoz et al., 2019). During the summer period, ${PM}_{2.5}$ concentrations generally get lower. During the transition period, high ${PM}_{2.5}$ concentrations are also found over the Benelux, the western part of Germany (Ruhr area) and major cities in Eastern Europe (up to ~ 20 μg/m3).

### 3.2 Linear component and chemical regimes

The overall linear component is approximatively equal to the sum of the potential impacts of each precursor computed for a 10% emission reduction (Eq. (3)). Non linearities for that level of reduction are homogeneous over Europe and everywhere below 4% (not shown). The three terms of the linear component ($P_{NH3}^{0.1}$, $P_{\mathrm{NOx}}^{0.1}$and $P_{SO2}^{0.1}$) are calculated in each cell of the model in order to compare the sensitivity of ${PM}_{2.5}$ concentrations to 10% reduction in ${NH}_{3}$, ${NO}_{x}$, and ${SO}_{2}$ emission reductions. When the reduction of ${NH}_{3}$ produces the largest impact on PM concentrations (i.e. $P_{NH3}^{0.1}>max\left(P_{\mathrm{NOx}}^{0.1};P_{SO2}^{0.1}\right)$) the chemical regime is called ${NH}_{3}$-sensitive. We do similarly for ${NO}_{x}$ and ${SO}_{2}$. We can then apply this calculation to each grid cell of the domain to delineate spatially the different chemical regimes.

**Fig. 2:**
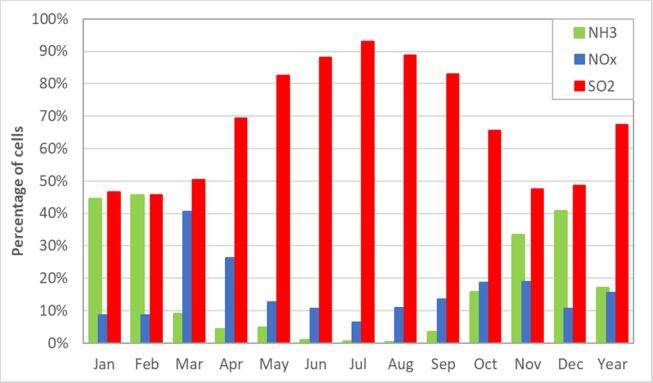
Percentage of cells (among all land grid cells within the entire domain) for each chemical regime, in green ${NH}_{3}$-sensitive, in blue ${NO}_{x}$-sensitive and in red ${SO}_{2}$-sensitive.

To determine representative time periods within the year for the temporal analysis, we compare domain-integrated statistics on a monthly basis. Only land-based grid cells are considered. Fig. 2 shows that the number of ${SO}_{2}$-sensitive cells dominates throughout the year with a strong increase between April to October to reach a maximum (more than 90% of the cells) in July. The number of ${NH}_{3}$-sensitive cells is high in winter (around 35 to 45% of the cells between November and February) but relatively low for the rest of year. Finally, the number of ${NO}_{x}$-sensitive cells is generally low (around 10%) but with two maxima: in spring (March and April) and in Autumn (October and November).

In Annex B, we show that the application of an ascending hierarchical classification method to the monthly cell percentage of the ${NO}_{x}$, ${NH}_{3}$, ${SO}_{2}$ dominating chemical regimes leads to the identification of three periods in the year: May to September (referred to as Summer in this work) when the number of ${SO}_{2}$-sensitive cells is above its yearly average value, November to February (Winter) when the number of ${NH}_{3}$-sensitive cells is above its yearly average value and, March, April and October (Transition) when the number of ${NO}_{x}$-sensitive cells is above its yearly average value.

**Fig. 3:**
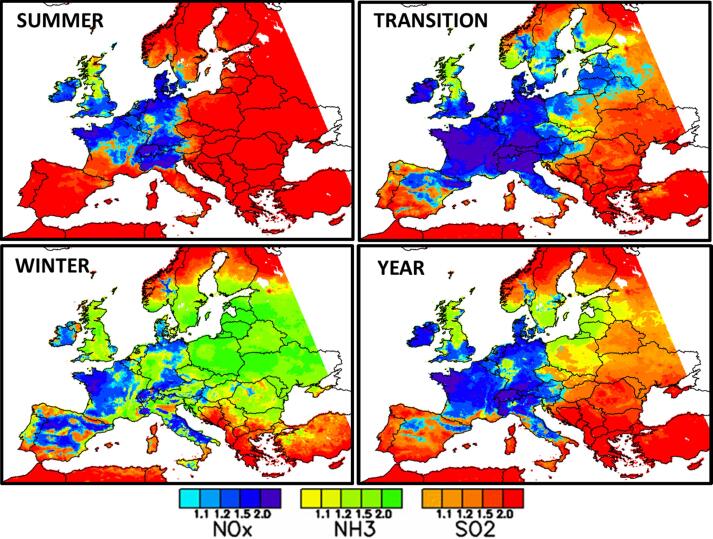
Spatial chemical regimes based on a 10% emission reduction of the ${NH}_{3}$, ${NO}_{x}$ and ${SO}_{2}$PM precursors. In yellow to green ${NH}_{3}$-sensitive, in light to dark blue ${NO}_{x}$-sensitive and in orange to red ${SO}_{2}$-sensitive. For each pollutant, the color scale differentiates different levels according to the difference between the top and second ranked potential impacts among the three precursors. The levels 1.1, 1.2, 1.5 and 2 correspond to differences of 10, 20, 50 and 100%, respectively.

Fig. 3 shows the spatial distribution of the dominating secondary ${PM}_{2.5}$ formation chemical regimes across Europe (in yellow to green ${NH}_{3}$-sensitive, in light to dark blue ${NO}_{x}$-sensitive and in orange to red ${SO}_{2}$-sensitive). In summer, the ${SO}_{2}$-sensitive regime dominates the largest part of Europe (all South and East of Europe). A large area including northern Italy, the western part of Austria, Switzerland, Germany, Denmark, northern France, Belgium, Netherlands, southern UK and Ireland is ${NO}_{x}$-sensitive. Only two small regions (Scotland (UK) and the Ruhr area (DE)) are ${NH}_{3}$-sensitive. During wintertime, the ${NH}_{3}$-sensitive regime area becomes more important and covers Eastern Europe, southern Scandinavia, UK, western Germany, eastern France, the western coast of Portugal, Sicily (IT) etc… ${NO}_{x}$-sensitive areas are observed in Ireland, central Spain, western France, central Italy and eastern Germany whereas a part of southern Europe (southern Spain, Mediterranean islands, Balkans) and northern Scandinavia remain ${SO}_{2}$-sensitive. During the Transition months, the ${NO}_{x}$-sensitive regime area expands to western Europe (Ireland, southern Britain, northern Spain, France, Belgium, Netherland, central and northern Italy, Switzerland, Austria, Germany, Denmark, southern Sweden, Czech Republic, western Poland, and the Baltic states). Southern, eastern and northern Europe (Portugal and southern Spain, Mediterranean islands, southern Italy, Balkans states and eastern European countries, northern Scandinavia) are under a ${SO}_{2}$-sensitive regime. The ${NH}_{3}$-sensitive regime spreads over northern UK, southern Poland, southern Norway and Finland.

When the relationship between emission and concentration is linear, potential impacts remain constant regardless the percentage reduction in emissions. In other words, the maps presented above would remain unchanged for other emission reduction strengths as far as linearity is preserved. It is therefore important to assess the degree of non-linearity and its implication in terms of distribution of chemical regimes.

### 3.3 Non-linearities

#### 3.3.1 The overall non-linearity in relative terms

The importance of non-linearity can be assessed through Eq. (2), expressed here as the sum of two percentages (10):

(10) $$\frac{P_{\mathrm{all}}^{\mathrm{lin}}}{P_{\mathrm{all}}^{\alpha }}\times 100+\frac{{\hat{P}}_{\mathrm{tot}}^{\alpha }}{P_{\mathrm{all}}^{\alpha }}\times 100=100$$

**Fig. 4:**
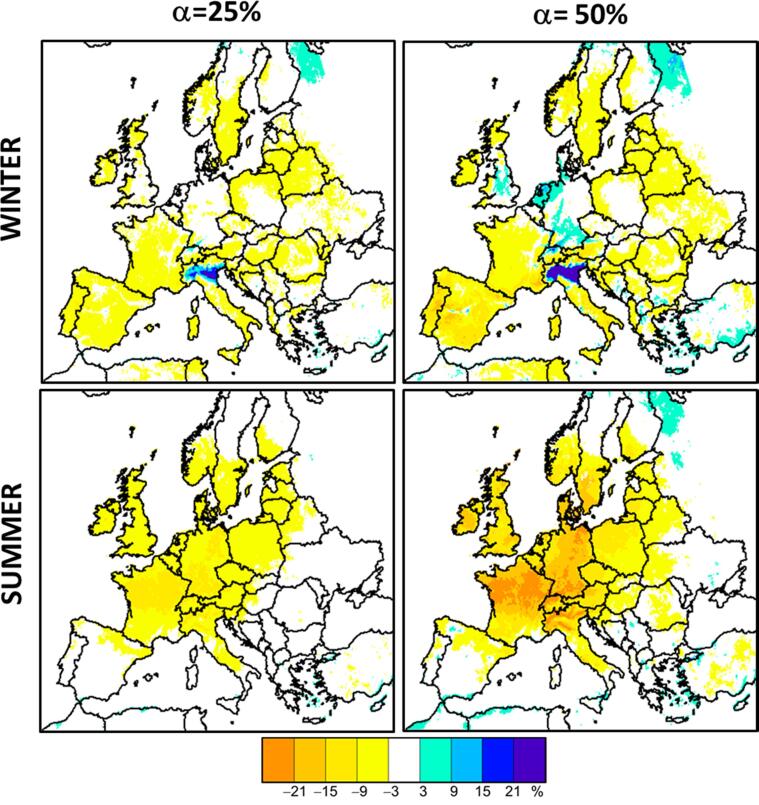
Percentage of non-linear deviation $\left({\hat{P}}_{\mathrm{tot}}^{\alpha }/P_{\mathrm{all}}^{\alpha }\times 100\right)$ for a 25% (left) and 50% (right) emission reduction computed compared to the 10% reduction level. For the 25% reduction, max and min percentages range between −16% to 11% in summer, and between −12% to 32% in Winter. For the 50% emission reduction, the corresponding min–max percentages are: −39% to 20% (Summer) and –22% to 63% (Winter).

In this expression, $P_{\mathrm{all}}^{\mathrm{lin}}/P_{\mathrm{all}}^{\alpha }$and ${\hat{P}}_{\mathrm{tot}}^{\alpha }/P_{\mathrm{all}}^{\alpha }$ represent the linear and non-linear fractions, respectively. Positive $\hat{P}$ mean that potential impact increases with emission reduction strength “in other worlds”, larger emission reductions will have proportionally more effect on concentrations (Annex A, case A), and reversely when $\hat{P}$ is negative (Annex A case B). In Fig. 4 left hand side, we see that for a 25% emission reduction, the non-linear fraction (non-linear deviation) is almost everywhere and every time negative and relatively low (less than 15%). The main exception is northern Italy (Po Valley region) that stands out with an average positive winter non-linear deviation term of the order of 20% but reaching up to 32% in some locations. The non-linearity fraction increases significantly when emissions are reduced by 50% (Fig. 4 right hand side). In Summer, a large area extending from Ireland to Germany, including France and the Benelux shows negative non-linearity values reaching −20%. In winter, the positive non-linearity reinforces below 20% within the Po Valley and new positive areas appear in the north of Switzerland, the Ruhr area in Germany, the Netherland, north-west of London in England, or in Madrid (Spain).

#### 3.3.2 Non-linear components

**Fig. 5:**
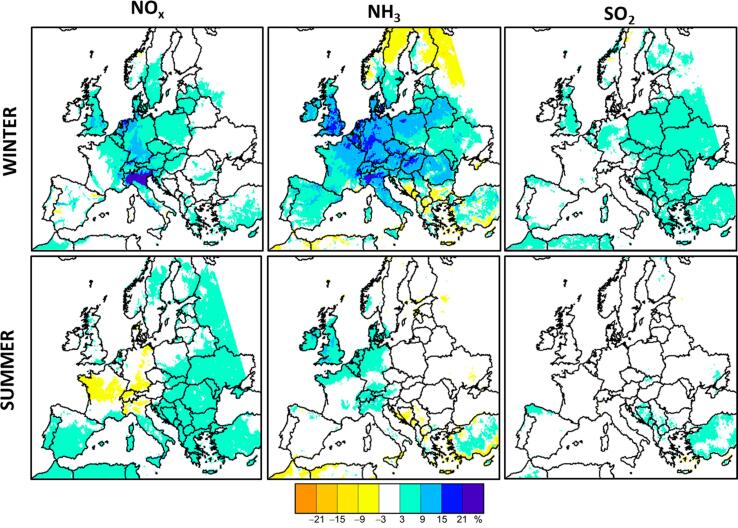
Single precursor non-linear components computed for a 50% emission reduction, ${\hat{P}}_{\mathrm{NOx}}^{0.5}/P_{\mathrm{all}}^{0.5}\times 100$ (left), ${\hat{P}}_{NH3}^{0.5}/P_{\mathrm{all}}^{0.5}\times 100$ (center), ${\hat{P}}_{SO2}^{0.5}/P_{\mathrm{all}}^{0.5}\times 100$ (right).

While in the previous section we assessed the overall level of non-linearity, we analyze here its components for the different modelling scenarios. Different types of non-linear components are identified in Eq. (4). We start here by analyzing the single precursor non-linearities (${\hat{P}}_{NH3}^{\alpha }/P_{\mathrm{all}}^{\alpha }\times 100$, ${\hat{P}}_{\mathrm{NOx}}^{\alpha }/P_{\mathrm{all}}^{\alpha }\times 100$, and ${\hat{P}}_{SO2}^{\alpha }/P_{\mathrm{all}}^{\alpha }\times 100$), shown in Fig. 5.

Areas with high single precursor non-linearity values indicate that potential impacts vary significantly with the emission reduction strength α (Eq. (5)). Different single non-linearities between precursors can therefore lead to changes in terms of secondary PM formation chemical regime. For example, the single non-linearity in Germany is larger for ${NH}_{3}$ than for ${NO}_{x}$ or ${SO}_{2}$, leading to regime changes from ${NO}_{x}$ to ${NH}_{3}$ sensitive in this region for larger emission reductions (see a more detailed analysis in the next section). More generally, single precursor non-linearity values inform on the robustness of the ${PM}_{2.5}$ responses to emission changes, with low and high values highlighting stable or variable chemical regimes, respectively.

The maps (Fig. 5) show that the single precursor non-linear components generally remain low (i.e. between −10% and 10%) in summer. They are however higher in winter, especially for the ${NO}_{x}$ component which reaches 21% in the Po Valley and ranges between 9 and 15% in the Ruhr area (Germany) and in the Netherlands. The ${NH}_{3}$ component ranges between 9 and 21% in a large area extending from Italy to UK and Denmark and from northern and eastern France to Romania and Belorussia.

**Fig. 6:**
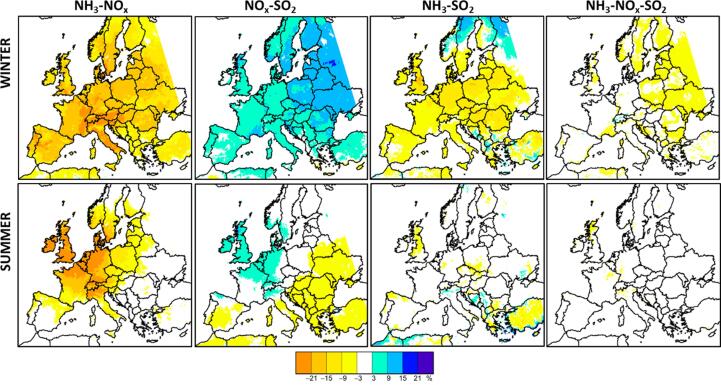
Non-linear components related to 2 and 3 precursors for a 50% emission reduction, ${\hat{P}}_{NH3-NOx}^{0.5}/P_{\mathrm{all}}^{0.5}\times 100$ (extreme left), ${\hat{P}}_{\mathrm{NOx}-SO2}^{0.5}/P_{\mathrm{all}}^{0.5}\times 100$ (center left), ${\hat{P}}_{NH3-SO2}^{0.5}/P_{\mathrm{all}}^{0.5}\times 100$ (center right), ${\hat{P}}_{NH3-NOx-SO2}^{0.5}/P_{\mathrm{all}}^{0.5}\times 100$ (extreme right).

On top of the single precursor non-linearities, Eq. (5) also includes multi-precursors non-linear interaction terms that can either involve two: ${\hat{P}}_{NH3-NOx}^{\alpha }/P_{\mathrm{all}}^{\alpha }$, ${\hat{P}}_{\mathrm{NOx}-SO2}^{\alpha }/P_{\mathrm{all}}^{\alpha }$, ${\hat{P}}_{NH3-SO2}^{\alpha }/P_{\mathrm{all}}^{\alpha }$ or three precursors (${\hat{P}}_{NH3-NOx-SO2}^{\alpha }/P_{\mathrm{all}}^{\alpha }$). All multi-precursors non-linear interaction components are generally negative with the exception of the ${NO}_{x}$-${SO}_{2}$ component during both seasons (Fig. 6). This means that the impact of reducing the emissions of both precursors is less than the sum of the impacts of reducing the emissions of one of them only. This result is expected because a reduction of only ${NO}_{x}$ implies a reduction of both ${NO}_{3}^{-}$ and ${NH}_{4}^{+}$ and the same happens when reducing only ${NH}_{3}$, therefore a simultaneous reduction of both precursors is lower than the sum of the two. Conversely, the ${NO}_{x}$-${SO}_{2}$ non-linear interaction term is positive in vast areas across Europe during both seasons. This indicates that the impact on ${PM}_{2.5}$ of reducing both ${SO}_{2}$ and ${NO}_{x}$ emissions would be larger than the sum of the impacts of ${NO}_{x}$ and ${SO}_{2}$ emission reduction only.

**Fig. A2:**
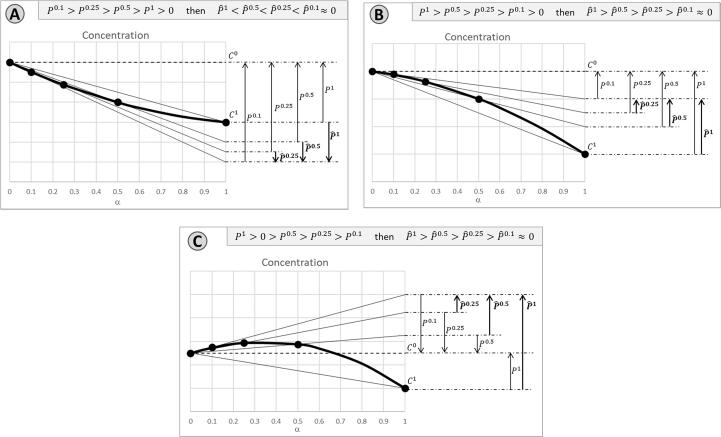
Evolution of the concentration v.s. the emission reduction strength in three main situations: A (top), B (center)and C (bottom).

It is worth noting that in most cases, the negative single non-linear components are compensated by the positive multi pollutant interaction terms. In winter, in Germany, both the ${NH}_{3}$ and ${NO}_{x}$ single components are positive (between 3 and 15% for ${NO}_{x}$ and between 9 and 15% for ${NH}_{3}$, Fig. 5) indicating that ${NO}_{x}$ and ${NH}_{3}$ potential impacts are slightly increasing with α in this area (Fig. A.2 center). However, this is compensated by the negative multi precursor component (especially the ${NH}_{3}$-${NO}_{x}$ interaction), resulting in very low overall non-linearities (i.e. less than 3%, Fig. 4). In other words, despite the changing ${NO}_{x}$ and ${NH}_{3}$ potentials, the overall potential (related to the reduction of all precursors) remains constant.

In previous works (Thunis et al., 2015, Pisoni et al., 2017) the authors quantified the non-linearity of model responses to emission reductions in different locations in Europe. One of their conclusions was that non-linearities remain relatively low for yearly averaged responses. The results presented here confirm these findings with the only exception of the Po basin area where important nonlinearities are modelled. However, these occur mainly during wintertime and are limited to specific areas within the Po basin. The peculiarity of the Po valley and the strong spatial variability characterizing the chemical regimes in this area are discussed in details in Thunis et al. (2021a).

### 3.4 Underlying chemical processes

#### 3.4.1 Chemical reactions

Inorganic aerosols are formed through different pathways (e.g. Seinfeld and Pandis, 2006). The formation of ammonium nitrate (${NH}_{4}{NO}_{3}$) in the particulate matter results from the gas phase combination of nitric acid ($HNO_{3}$) and ammonia ($NH_{3}$) via (R3). Nitric acid itself can be produced from ${NO}_{2}$through two pathways. The first (R1) requires OH radicals while the second (R2) requires both $O_{3}$ and a wet aerosol surface.

(R1) $${NO}_{2}\left(g\right)+OH+M\to {HNO}_{3}\left(g\right)+M$$

(R2) $$\left(\begin{array}{c} {NO}_{2}\left(g\right)+O_{3}\left(g\right)\to {NO}_{3}\left(g\right)+O_{2}\left(g\right)\\ {NO}_{3}\left(g\right)+{NO}_{2}\left(g\right)↔N_{2}O_{5}\left(g\right)\\ N_{2}O_{5}\left(g\right)+H_{2}O\left(aerosolsurface\right)\to 2HNO_{3}\left(g\right) \end{array}\right\}$$

(R3) $${HNO}_{3}\left(g\right)+NH_{3}\left(g\right)↔{NH}_{4}^{+}\left(p\right)+{NO}_{3}^{-}\left(p\right)$$

Particulate ammonium sulfate (${\left(NH_{4}\right)}_{2}SO_{4}$) results from the combination of sulfuric acid ($H_{2}{SO}_{4}$) already present in the particulate phase and gas phase ammonia ($NH_{3}$) via R6. The character →→ is used to symbolize a chemical pathway that summarizes a set of underlying reactions.

(R4a) $${SO}_{2}\left(g\right)+OH\to \to H_{2}{SO}_{4}\left(g\right)$$

(R4b) $${SO}_{2}\left(aq\right)+O_{3},H_{2}O_{2}\left(aq\right)\to \to H_{2}{SO}_{4}\left(aq\right)$$

(R5) $$H_{2}{SO}_{4}\left(g\right)\to H_{2}{SO}_{4}\left(p\right)\to {2H}^{+}\left(p\right)+{SO}_{4}^{2-}\left(p\right)$$

(R6) $$H_{2}{SO}_{4}\left(p\right)+2NH_{3}\left(g\right)\to 2{NH}_{4}^{+}\left(p\right)+{SO}_{4}^{2-}\left(p\right)$$

In these reactions, $\left(g\right)$ means “gas phase”, (aq) refers to the liquid phase (rain drops or cloud droplets), while (p) refers to aerosol particles which can be solid or liquid but are always smaller than cloud droplets and rain drops.

The main characteristics of the nitrate (${NO}_{3}^{-}$) and sulfate (${SO}_{4}^{2-}$) formation can be summarized in the two following points:

1. The formation of inorganic salts from ${SO}_{2}$ and ${NO}_{2}$ oxidation depends on the concentration of oxidants in the atmosphere to yield condensable products such as $H_{2}{SO}_{4}$ and $HNO_{3}$. The greater the oxidizing capacity of the atmosphere is, the more efficient the formation of nitrate and sulfate will be. The oxidizing capacity of the atmosphere is high when the concentration of radicals (such as OH) and photooxidants (such as $O_{3}andH_{2}O_{2}$) is high.
2. While the phase transition of gaseous the ${HNO}_{3}$ to particulate nitrate requires ammonia (which can become a limiting factor), this is not the case for particulate sulfate that can be formed from ${SO}_{2}$ oxidation only.

#### 3.4.2 “Most common situations”

**Fig. C1:**
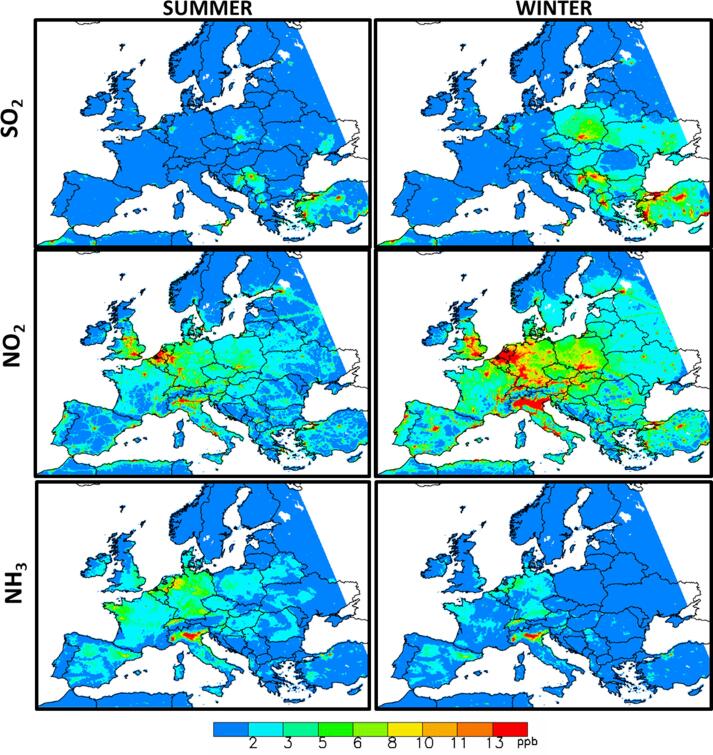
Maps of ${SO}_{2}$, ${NO}_{2}$ and ${NH}_{3}$ in summer and winter.

Case 1: Wintertime $NH_{3}$-limited regime:

In winter, large $NH_{3}$-sensitive areas are modelled in Eastern European countries, south Scandinavia and in the UK (Fig. 3). In these areas the overall non-linearity is very low (Fig. 4) and dominated by ${NO}_{x}$-$NH_{3}$ interactions (Fig. 5 left). $NH_{3}$ concentrations are relatively low (agriculture emits less ammonia during winter) (Fig. C.1). In contrast, ${NO}_{x}$ concentrations are high because of the reduced vertical dispersion and increased ${NO}_{x}$ emissions (due to domestic heating demand for example) (Fig. C.1).

In addition, $SO_{2}$ is also large (Fig. C.1) and subsequent ${H_{2}SO}_{4}$ will bind part of ${NH}_{3}$ as $NH_{4}^{+}$. The chemical regime is rather linear with respect to $NH_{3}$ emission reductions as long as enough free NH_X_, not bound by ${H_{2}SO}_{4}$, is available.

**Fig. 7:**
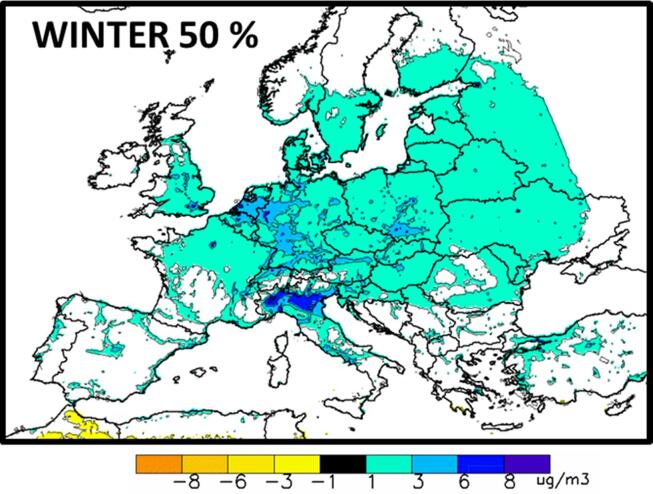
$O_{3}$ wintertime concentration changes (Base Case – Scenario) resulting from a 50% reduction of the ${NO}_{x}$ emissions.

Case 2: Wintertime positive non-linearity:

In winter, a strong positive non-linearity (Fig. 4), dominated by the ${NO}_{x}$ component (Fig. 5) is calculated for several areas (the Po Valley, north of Switzerland, Ruhr area in Germany, The Netherlands, north-east of London in UK, Madrid in Spain). All these areas are characterised by large ${NO}_{x}$ emissions. In terms of pollution, the Po Valley is certainly the worst case at least partly because it is surrounded by two mountain ranges (the Alps to the west and north and the Apennines to the south) that reduce the dispersion of pollutants (De Meij et al., 2009b). Although $O_{3}$ does not generally represent a regulatory issue (no threshold exceedances) during wintertime, it plays an important role in the ${PM}_{2.5}$ response to ${NO}_{x}$ emission reductions. The regions of strong positive non-linearity correspond to VOC limited $O_{3}$ formation regime areas, where ${NO}_{x}$ emission reductions lead to significant increases in $O_{3}$ concentrations (Fig. 7) due to a decreased titration of $O_{3}$ by NO. However, ${NO}_{x}$ emission reductions also lead to lower ${NO}_{2}$ concentrations. In such a situation, two competing phenomena occur: the reduction ${NO}_{2}$ concentrations tend to decrease the ${HNO}_{3}$ production, while the augmentation in oxidant concentrations tends to increase the ${HNO}_{3}$ production (R1, R2). These two competing effects then result in the reduction of the ${HNO}_{3}$ production and, therefore, of nitrate concentration (R3). But this reduction is however much lower than it would be if the ${NO}_{2}$ emission - nitrate concentration relationship were proportional. The increase in the oxidizing capacity of the atmosphere also leads to an increase in $H_{2}{SO}_{4}$ production through its aqueous phase pathway formation (R4b), which is dominant in winter, and to an increase in sulfate (R5). As a result of these reactions, ${NO}_{x}$ emission reductions lead to a positive non-linearity: the stronger the ${NO}_{x}$ emission reductions are, the stronger the potential impacts on nitrate and sulfate are. This is explained by the fact that for small ${NO}_{x}$ emission reductions, the subsequent $O_{3}$ and related oxidant concentration increase prevents SIA formation reduction, while for stronger ${NO}_{x}$ emission reductions, the effect on $O_{3}$ and oxidants becomes less and less important and nitrate formation reduction due to the decrease in ${NO}_{x}$ concentration takes over. This mechanism is mostly pronounced within the Po basin (see a more in-depth discussion in Thunis et al., 2021a), but also occurs to a lesser degree over Western Germany and The Netherlands.

Case 3: Summer time negative non-linearity:

In summer, northwestern and central Europe are characterized by a ${NO}_{x}$ sensitive regime (Fig. 3), and a strong negative overall non-linearity (Fig. 4) dominated by the ${NO}_{x}$-$NH_{3}$ component (Fig. 5). This negative non-linearity is linked to (R3) in which both $NH_{3}$ and ${HNO}_{3}$ concentrations interfere. It indicates that the sum of the $NH_{4}NO_{3}$ concentration decrease resulting from $NH_{3}$ or ${NO}_{2}$ emission reductions alone has a larger impact than a simultaneous reduction of both precursors’ emission, suggesting that the $NH_{4}NO_{3}$ formation is not simply limited by one of the two precursors ${NO}_{2}$ or $NH_{3}$.

**Fig. C2:**
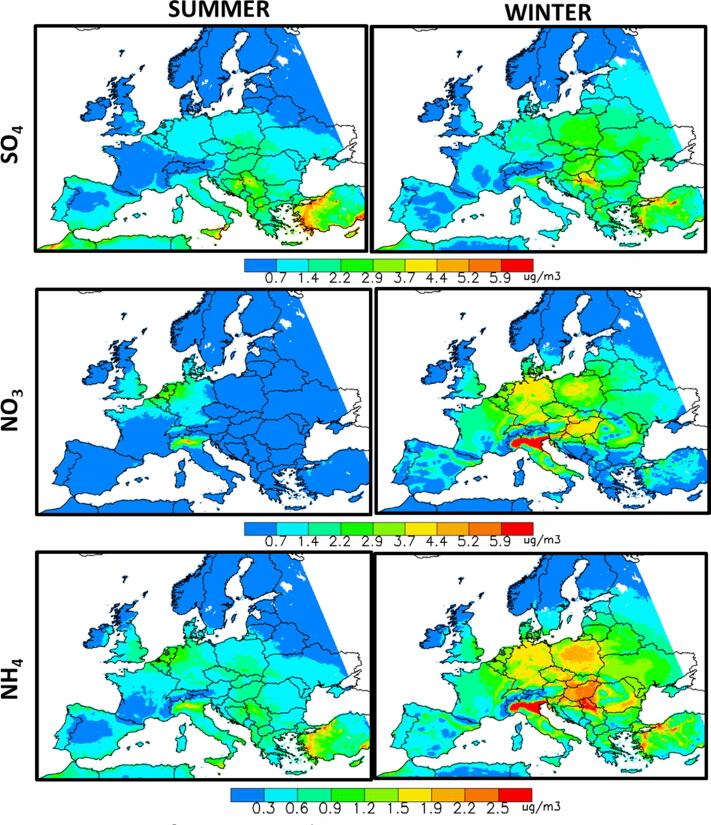
Maps of ${SO}_{4}^{2-}$, ${NO}_{3}^{-}$ and ${NH}_{4}^{+}$ in summer and winter.

Case 4: Summer $SO_{2}$-sensitive areas:

In summer, southern, eastern and northern Europe (Spain, south and central Italy, Balkans, central and eastern European countries, Scandinavia) is characterized by a ${SO}_{2}$-sensitive regime (Fig. 3) and a weak overall negative non-linearity (Fig. 4). The ${SO}_{2}$ contribution to the formation of inorganic particulate matter in these regions is major (Fig. C1, Fig. C2). Given that particulate sulfate formation is not limited by $NH_{3}$(reactions R4-R5), little or no non-linearities are modelled for ${SO}_{2}$ emission reduction impacts on ${PM}_{2.5}$ concentrations. Moreover, during summer, oxidant levels are only little affected by ${SO}_{2}$ oxidation, thus leading to a first order relationship between ${PM}_{2.5}$ concentration and ${SO}_{2}$ emission reductions.

## 4 Implication for policy making

Air quality models suffer from well-known weaknesses that result from their simplified representation of the complex reality (some processes remain unknown or not known enough to be captured in models). Despite these weaknesses, models represent the only option to design strategies where the impact of potential air quality measures needs to be assessed. But it is challenging to advice decision-makers effectively when model diagnostics are not robust, i.e. if model diagnostics change significantly when different input parameters are used (e.g. different levels of the emission reductions). As decisions must be efficient, it is crucial to assess the robustness associated to modelling results, in particular when non-linear effects of emission reductions under variable chemical regimes have been highlighted, as discussed above. In this section we discuss this robustness with respect to space, time and emission reduction strength.

### 4.1 Spatial and temporal robustness

As seen earlier, the chemical regimes map (Fig. 3) highlights an important spatial variability in ${PM}_{2.5}$ sensitivity to SIA precursor emission reductions (example Spain in Winter where the three chemical regimes occur within distance of few hundred km). The maps also show an important temporal variability, with only few areas keeping the same regime from Summer to Winter (for example Britany in France). The spatial and temporal distribution of the chemical regimes is therefore highly variable in Europe.

### 4.2 Robustness with regards to the reduction percentage

**Fig. 8:**
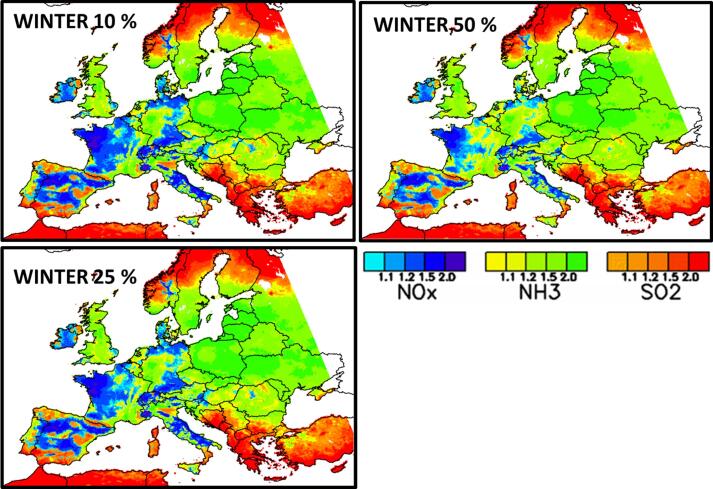
Winter chemical regimes computed for the 10, 25 and 50% emission reduction. For each pollutant, the color scale differentiates different levels according to the difference between the top and second ranked potential impacts among the three precursors. The levels 1.1, 1.2, 1.5 and 2 correspond to differences of 10, 20, 50 and 100%, respectively.

Table 1 provides an overview of the number of cells (in percentage values) that switch chemical regime when emission reduction strengths are changing (from 10% to 25% and from 10 to 50%). We see that the number of shifted cells:

• is larger for more intense emission reductions (i.e. the number of changing cells is larger from 10 to 50% than from 10 to 25%).
• is systematically larger in Winter than in Summer
• is always low (less than 10%) except for ${NO}_{x}$-sensitive regime cells when it reaches 30% in Winter. As visible from Fig. 8 (e.g. region Eastern Germany) the ${NO}_{x}$-sensitive cells almost exclusively shift to $NH_{3}$-sensitive.

**Table 1:** Percentage of domain grid cells that shift chemical regime when emission reductions increase from 10 and 25% (first data column) and from 10 to 50% (second data column). Percentages are expressed relative to the total number of cells in one given regime while the absolute number of cells is provided within square brackets. Only land grid cells are used in the statistics.

		10% → 25%		10% → 50%	
**Summer**	NH_3_ sensitive	1.0%	[23]	5.1%	[119]
	SO_x_ sensitive	0.04%	[78]	0.1%	[193]
	NO_x_ sensitive	3.0%	[445]	9.4%	[1390]
	**Total**	0.5%	[546]	1.5%	[1702]
**Winter**	NH_3_ sensitive	1.0%	[465]	3.9%	[1903]
	SO_x_ sensitive	0.3%	[501]	0.7%	[1091]
	NO_x_ sensitive	8.1%	[1207]	30.1%	[4497]
	**Total**	1.9%	[2173]	6.4%	[7491]

The spatial distribution of the chemical regimes computed in Winter for the 10, 25 and 50% emission reduction scenarios are plotted in Fig. 8. The chemical regimes remain similar in a large part of Europe (Central and southern Italy, Spain, Portugal, western France, Ireland, UK, Scandinavia, eastern Europe, Greece and Turkey) whereas they are changing in a few areas: When going from 10 to 50% emissions reductions, the ${NO}_{x}$-sensitive regime is mostly replaced by a $NH_{3}$-sensitive regime in the north and east of France, central Germany and Denmark and the ${SO}_{2}$-sensitive regime evolves into a ${NH}_{3}$- and ${NO}_{x}$-sensitive regimes in the northern part of Italy (Po Valley). Unlike their spatial and temporal low robustness, the chemical regimes are quite robust in terms of emission reduction strength (at least up to 50% emission reductions).

### 4.3 Implications on the design of strategies

**Fig. 9:**
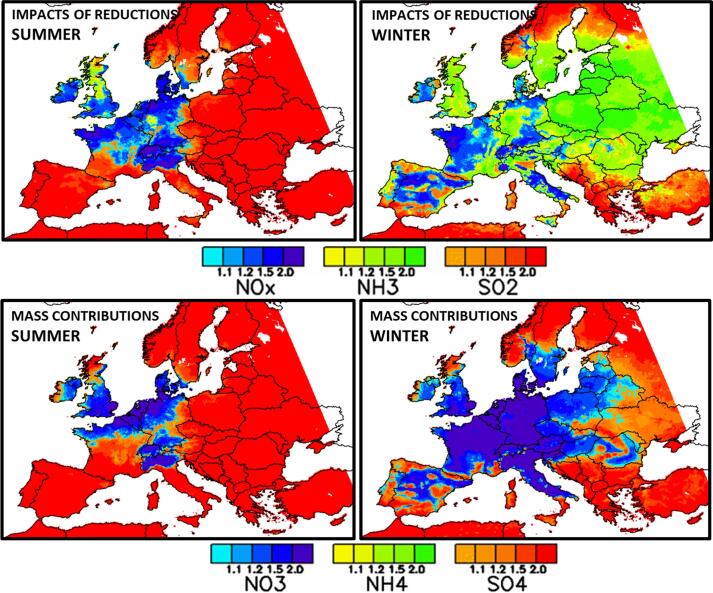
Top: chemical regimes distinguishing the emission precursor ($NH_{3}$ in green, ${SO}_{2}$ in red, $NO_{x}$ in blue) leading to the largest PM reduction for 10% emission reductions (same as Fig. 3) – Bottom: species which most contributes to PM_2.5_ concentrations using a tagging/labelling approach ($NH_{4}$ in green, ${SO}_{4}$ in red, $NO_{3}$ in blue). For each pollutant, the color scale differentiates different levels according to the difference between the top and second ranked potential impacts among the three precursors. The levels 1.1, 1.2, 1.5 and 2 correspond to differences of 10, 20, 50 and 100%, respectively.

The chemical regimes maps (Fig. 9 **–** top) provides valuable information to design strategies by orienting the choice on the precursor(s) whose emission reduction leads to the largest PM concentration decrease. While results are quite robust in terms of emission reduction intensity (secondary PM formation chemical regimes remain quite similar up to 50% emission reductions), this is not true in terms of time and space. The spatial and temporal variabilities of these chemical regimes point to the need of considering specific analysis in terms of regions and seasons. This is an important consideration as EU policies are usually driven by overall objectives (EU National Emissions Ceilings Directive, NEC, 2016) that translate into emission reduction strategies defined at the country level. In this context, Peduzzi et al. (2018) showed that higher resolution strategies, addressing in particular the specificities of cities, are more effective.

Intuitively, we would expect that the more abundant a PM constituent is compared to the others, the more effective the reduction of its precursors will be on PM levels. To check this intuitive reasoning, it is interesting to compare the chemical regimes maps discussed so far (Fig. 9 – top) to maps that are based on mass contributions via labeling/tagging techniques (Fig. 9 – bottom). Tagging/labelling techniques are implemented into air quality models to estimate the pollutant mass transferred from different emission sources to the ambient concentration at a given location and for a given time period (Thunis et al., 2019). We see that ${SO}_{2}$ behaves as expected. In summer, with few exceptions (Scotland and the Ruhr area), the areas where sulfate mass is the highest (Fig. 9 **–** bottom left) are very similar to the those where ${SO}_{2}$ reductions generate the largest PM reductions (Fig. 9 **–** top left). This is explained by the fact that the sulfate production from ${SO}_{2}$ mainly results from first-order processes (reactions depends only one reactant).

The situation is radically different for $NH_{3}$ and winter. Areas where $NH_{3}$ reductions are the most effective (Fig. 9 – top right) do not correspond to the areas where the ammonium mass is the largest (Fig. 9 – bottom left). This can be explained by the formation process of nitrate from $NO_{2}$ that also requires $NH_{3}$ and by the fact that the molecular weight of $NH_{4}$ (18 g.mol^−1^) is much lower than the one of $NO_{3}$ (62 g.mol^−1^). In winter, the low $NH_{3}$ emissions combined with the large $NO_{2}$ emissions lead to a $NO_{2}$ saturated nitrate production therefore limited by $NH_{3}$. Reductions of $NO_{2}$ concentrations have therefore a low impact, in contrast to $NH_{3}$ reductions that are very effective. Thus, tagging and sensitivity simulations give very different responses, for a case where a target species depends on several precursors in a non-linear manner.

Situations characterized by a $NH_{3}$-limited formation of secondary PM perfectly highlight complex processes which are not captured by the tagging approaches, but must be considered when designing air quality strategies. One of the implications of this finding is that methodologies that produce source apportionment on the basis of the mass or mole contributions (Tagging methods) are not suitable for predicting the impacts of emission reduction scenarios. The results produced by these methods can be misleading and target to the wrong precursor, e.g. decrease in priority $NO_{x}$ instead of $NH_{3}$ emissions (Thunis et al., 2019, Clappier et al., 2017). It also appears that emission reduction measures targeting $NH_{3}$ are less efficient during summer when $NH_{3}$ emissions are the largest, but conversely very efficient during winter when $NH_{3}$ emissions are already low.

In terms of implications on PM abatement strategies, our findings can be summarized in the following key messages: (1) reducing ${SO}_{2}$ emissions where ${SO}_{2}$ is abundant is always efficient as the relationship between ${SO}_{2}$ emissions and sulfate concentration is quite linear (e.g. eastern Europe and Balkans); (2) reducing $NH_{3}$ emissions where $NH_{3}$ is abundant can have a positive but limited effect, because secondary inorganic PM formation is little sensitive to $NH_{3}$ (e.g. the Po basin); (3) conversely, reducing $NH_{3}$ emissions is most efficient when and/or where $NH_{3}$ emissions are low, as $NH_{3}$ then becomes the limiting factor in secondary inorganic PM formation; (4) reducing $NO_{x}$ emissions where $NO_{x}$ are abundant can be counter-productive, with potential increases of secondary PM formation due to the increased oxidizing capacity of the atmosphere; (5) as regions where both the $NH_{3}$ and $NO_{x}$ sensitive chemical regimes prevail are mixed within countries, both need to be targeted together, as pollution reduction policies need are defined at least at national levels. (6) measures should primarily target wintertime $NH_{3}$ practices. There is no seasonal distinction to be made regarding $NO_{x}$ emissions.

The points mentioned above focus on the expected gains in terms of PM_2.5_ concentrations. As noted, some of these might be achieved at the expense of increases of other pollutants like $NO_{2}$ or $O_{3}$. It is therefore essential to account for all these aspects when designing air quality strategies.

## 5 Conclusion

Given the remaining air quality issues in many European regions, smart air quality strategies are necessary to reduce the burden of poor air quality. While designing effective strategies for non-reactive primary pollutants is straightforward, this is not the case for secondary pollutants for which the relationship between the precursors’ emission changes and the resulting concentration changes can be nonlinear. Under such conditions, strategies targeting the largest emitting sources might not be the most effective.

In this work, we provide elements to better understand the role of heavily emitted air pollutants (${SO}_{2}$, $NO_{x}$, $NH_{3}$) on the formation of secondary inorganic aerosols (SIA). By quantifying PM_2.5_ concentration sensitivity to the emission reductions of each of these three precursors, we define and map various secondary PM_2.5_ formation chemical regimes across Europe. The main aim was to provide insight on the key precursors to target for designing effective air quality strategies. For this, we performed a series of specific simulations in which the emissions of all 3 SIA precursors were reduced together or not by −10% to −50%.

The analysis of the SIA formation chemical regimes and linear term magnitude maps allowed us to identify typical locations and situations. During wintertime, PM_2.5_ concentrations are predominantly $NH_{3}$-sensitive in the major part Europe, except in the eastern Mediterranean basin (${SO}_{2}$-sensitive), and in various regions of countries including Ireland, Denmark, France, Germany, Switzerland, Spain and Italy ($NO_{x}$-sensitive). The potential impact of emission reductions generally decreases with increasing emission reduction levels (negative non-linearity), except in areas including the Po Valley, northern Switzerland and The Netherlands, where positive non-linearity is due to the fact that marginal $NO_{x}$ emission reductions have little impact on PM_2.5_ concentrations in those regions. During summertime, PM_2.5_ are predominantly ${SO}_{2}$-sensitive in most of Europe, except in the north of Britain (NH_3_-sensitive), and in the remainder of northwestern Europe, roughly from Ireland to Germany and from Denmark to northern Italy ($NO_{x}$-sensitive). In almost the whole of Europe (except e.g. the Iberian Peninsula and the Balkans), the potential impact of emission reductions decreases with increasing emission reduction levels (relatively high negative non-linearity). While chemical regimes greatly vary in terms of time (seasonality) and in space (between different locations in Europe), these regimes remain quite independent from the emission reduction strengths (up to the −50% level considered in this work). This is an important finding that has implications on air quality strategies.

The $NH_{3}$-sensitive chemical regime leads to a situation that could seem paradoxical. Indeed, it appears mainly during wintertime, while $NH_{3}$ emissions are already low. Still, further reducing NH_3_ emissions during wintertime is the most efficient way for decreasing PM concentrations, and wintertime-specific abatement strategies should therefore target $NH_{3}$ emissions, when these emissions are the lowest. In such situations, we showed that methods based on precursor mass contribution (tagging) would not provide reliable information to design reduction strategies as they would wrongly target the precursor in excess while the less abundant precursor is the key. The results have also triggered some further questions, in particular on the importance of regulating $NH_{3}$ concentrations to support effective PM abatement strategies. Under such situations, $NO_{x}$ abatements would need to be very large to be effective, in fact large enough to shift chemical regime (from $NH_{3}$- to $NO_{x}$ sensitive)?

We also found that (1) reducing ${SO}_{2}$ emissions where ${SO}_{2}$ is abundant is always efficient as the relationship between ${SO}_{2}$ emissions and sulfate concentration is quite linear; (2) reducing $NO_{x}$ emissions where $NO_{x}$ are abundant can be counter-productive, with potential increases of secondary PM formation due to the increased oxidizing capacity of the atmosphere. As regions where both the $NH_{3}$ and $NO_{x}$ sensitive chemical regimes prevail are mixed within countries, both need to be targeted together, as pollution reduction policies need are defined at least at national levels.

The results obtained in this work were obtained with one model only, namely the EMEP rv_33 model. It would be important in the future to assess the robustness of these results by comparing them to other available air quality model outputs. The simulations proposed in this work could be used for benchmarking other models, since they constitute the type of scenarios required to support air quality strategies. In addition, the straight and systematic emission reductions imposed for the scenarios in this work are well suited for a better understanding of the behavior of models, in terms of concentration responses to emission reductions.

Finally, while emission reductions have been imposed Europe wide and whole year long, it might be of interest to check whether the same conclusions hold when emission reductions are applied to smaller domain (e.g. a region) or for a shorter time period (episode).

## Declaration of Competing Interest

The authors declare that they have no known competing financial interests or personal relationships that could have appeared to influence the work reported in this paper.

## Figures and Tables

**Fig. A1:**
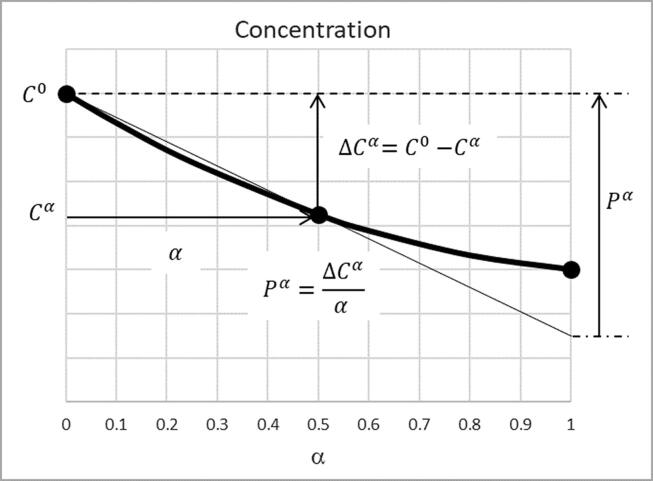
Concentration v.s. emission reduction strength (α).

**Fig. B1:**
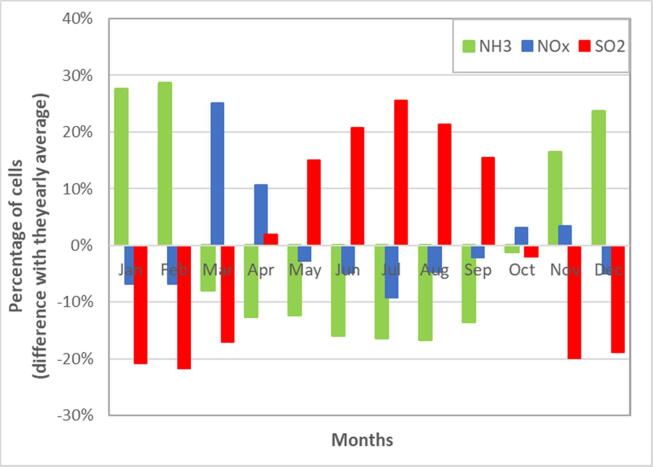
Difference between monthly and yearly percentages of cell with a given chemical regime. (${NH}_{3}$-sensitive in green, ${NO}_{x}$-sensitive in blue, ${SO}_{2}$-sensitive in red).

**Fig. B2:**
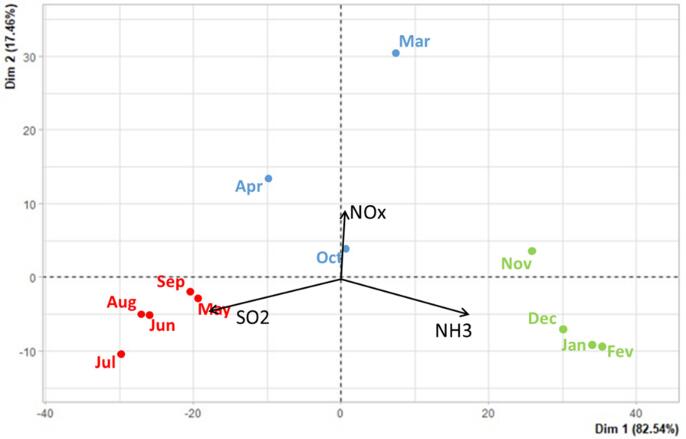
Repartition of the months according to the two first axes of the principal component analysis (PCA). The months are distributed by a bottom-up hierarchical cluster analysis into 3 groups: In green months where the percentages of ${NH}_{3}$-sensitive cells are above their yearly average, in red the months where the percentages of ${SO}_{2}$-sensitive cells are above their yearly average and in blue the months where the percentages of ${NO}_{x}$-sensitive cells are above their yearly average.

## Annex A From concentration changes to potential impacts: Decomposition and non-linearity

**Concentration changes**

We follow the formulation proposed by Clappier et al. (2017) adapted from Stein and Alpert (1993) that decomposes the concentration change resulting from the reduction of several precursors into a sum of terms that depend on one precursor only and of multi-precursor interaction terms. For two precursors (e.g. ${NH}_{3}$ and ${NO}_{x}$) it leads to:

(A.1) $$\Delta C_{NH3-NOx}^{\alpha }=\Delta C_{NH3}^{\alpha }+\Delta C_{\mathrm{NOx}}^{\alpha }+{\hat{C}}_{NH3-NOx}^{\alpha }$$

with α the emission reduction strength (varying from 0 to 1).

$\Delta C_{NH3-NOx}^{\alpha }$ the concentration change resulting from a simultaneous reduction of ${NH}_{3}$ and ${NO}_{x}$. $\Delta C_{NH3-NOx}^{\alpha }=C^{0}-C_{NH3-NOx}^{\alpha }$ where $C^{0}$ and $C_{NH3-NOx}^{\alpha }$ are the base case and emission reduction scenario PM concentrations, respectively.
$\Delta C_{NH3}^{\alpha }$; $\Delta C_{\mathrm{NOx}}^{\alpha }$ are the concentration changes resulting from the single precursor reductions. $\Delta C_{\mathrm{NOx}}^{\alpha }=C^{0}-C_{\mathrm{NOx}}^{\alpha }$ and $\Delta C_{NH3}^{\alpha }=C^{0}-C_{NH3}^{\alpha }$ where $C^{0}$, $C_{\mathrm{NOx}}^{\alpha }$ and $C_{NH3}^{\alpha }$ are the base case and reduction scenarios PM concentration, respectively.
${\hat{C}}_{NH3-NOx}^{\alpha }$ cannot be attributed to a specific precursor as it results from a combined effect of the emission reduction of several precursors. It is computed as the difference between the concentration change due to the simultaneous reduction of all precursors and the sum of the single precursor concentration changes:

(A.2) $${\hat{C}}_{NH3-NOx}^{\alpha }=\Delta C_{NH3-NOx}^{\alpha }-\Delta C_{NH3}^{\alpha }-\Delta C_{\mathrm{NOx}}^{\alpha }$$

The formulation (A.1), (A.2) can be generalized to more than 2 precursors, e.g. the 3 precursors: ${NH}_{3}$ and ${NO}_{x}$ and ${SO}_{2}$, as follows:

(A.3) $$\Delta C_{\mathrm{all}}^{\alpha }=\Delta C_{NH3}^{\alpha }+\Delta C_{\mathrm{NOx}}^{\alpha }+\Delta C_{SO2}^{\alpha }+{\hat{C}}_{\mathrm{all}}^{\alpha }$$

with $\Delta C_{\mathrm{all}}^{\alpha }$ the concentration change resulting from the simultaneous reduction of all precursors ${NH}_{3}$, ${NO}_{x}$ and ${SO}_{2}$.

${\hat{C}}_{\mathrm{all}}^{\alpha }$ quantifies the overall non-linear interactions and is computed as:

(A.4) $${\hat{C}}_{\mathrm{all}}^{\alpha }=\Delta C_{\mathrm{all}}^{\alpha }-\Delta C_{NH3}^{\alpha }-\Delta C_{\mathrm{NOx}}^{\alpha }-\Delta C_{SO2}^{\alpha }$$

${\hat{C}}_{\mathrm{all}}^{\alpha }$ can be decomposed into terms that relate to specific double or triple interactions among precursors:

(A.5) $${\hat{C}}_{\mathrm{all}}^{\alpha }={\hat{C}}_{NH3-NOx}^{\alpha }+{\hat{C}}_{NH3-SO2}^{\alpha }+{\hat{C}}_{\mathrm{NOx}-SO2}^{\alpha }+{\hat{C}}_{NH3-NOx-SO2}^{\alpha }$$

The double interaction terms can be computed in a similar way to (A.2):

(A.6) $${\hat{C}}_{NH3-NOx}^{\alpha }=\Delta C_{NH3-NOx}^{\alpha }-\Delta C_{NH3}^{\alpha }-\Delta C_{\mathrm{NOx}}^{\alpha }$$

(A.7) $${\hat{C}}_{NH3-SOx}^{\alpha }=\Delta C_{NH3-SO2}^{\alpha }-\Delta C_{NH3}^{\alpha }-\Delta C_{SO2}^{\alpha }$$

(A.8) $${\hat{C}}_{\mathrm{NOx}-SOx}^{\alpha }=\Delta C_{\mathrm{NOx}-SO2}^{\alpha }-\Delta C_{\mathrm{NOx}}^{\alpha }-\Delta C_{SO2}^{\alpha }$$

While the triple interaction term is computed as:

(A.9) $${\hat{C}}_{NH3-NOx-SO2}^{\alpha }={\hat{C}}_{\mathrm{all}}^{\alpha }-\Delta C_{NH3-NOx}^{\alpha }-\Delta C_{NH3-SO2}^{\alpha }-\Delta C_{\mathrm{NOx}-SO2}^{\alpha }$$

**Potential impacts**

Formulation (A.3) can be modified to obtain potential impacts, defined by Thunis et al. (2020) as the ratio between the concentration change and the emission reduction strength. Dividing all terms in A.3 by α, we obtain:

(A.10) $$P_{\mathrm{all}}^{\alpha }=\frac{\Delta C_{\mathrm{all}}^{\alpha }}{\alpha };P_{NH3}^{\alpha }=\frac{\Delta C_{NH3}^{\alpha }}{\alpha };P_{\mathrm{NOx}}^{\alpha }=\frac{\Delta C_{\mathrm{NOx}}^{\alpha }}{\alpha };P_{SO2}^{\alpha }=\frac{\Delta C_{SO2}^{\alpha }}{\alpha }$$

Potential impacts for a reduction α can be interpreted in two ways:

1) as the opposite of the average concentration slope between α and the baseline (α=0);
2) as the concentration change that would potentially result from a 100% reduction (α=1) if impacts were to be extrapolated linearly from α.

Similarly, to concentration deltas, potential impacts can be added and Eq. (A.3) expressed in terms of potentials would be:

(A.11) $$P_{\mathrm{all}}^{\alpha }=P_{NH3}^{\alpha }+P_{\mathrm{NOx}}^{\alpha }+P_{SO2}^{\alpha }+{\hat{P}}_{\mathrm{all}}^{\alpha }$$

Thanks to (A.9) the interaction terms can also be computed for potential impacts:

(A.12) $${\hat{P}}_{\mathrm{all}}^{\alpha }={\hat{P}}_{NH3-NOx}^{\alpha }+{\hat{P}}_{NH3-SO2}^{\alpha }+{\hat{P}}_{\mathrm{NOx}-SO2}^{\alpha }+{\hat{P}}_{NH3-NOx-SO2}^{\alpha }$$

**Linearity**

The potential impact is an indicator that is particularly relevant to assess the degree of linearity between emission changes and concentration changes. Indeed, potential impacts fulfill two properties when the relationship is linear:

1. Consistency: each precursor’s potential impact remains constant over the entire range of emission reductions: $\forall \alpha P_{NH3}^{\alpha }=const,P_{\mathrm{NOx}}^{\alpha }=const,P_{SO2}^{\alpha }=const$. When linearity applies, the potential impact for a given precursor is therefore equal to the concentration change resulting from a 100% reduction of that precursor (α=1).

2. Additivity: For any reduction percentage, the sum of the single concentration changes equals the concentration change resulting from the simultaneous reduction of all precursors. This implies that ${\forall \alpha \hat{P}}_{\mathrm{all}}^{\alpha }$ = 0.

Note that linearity holds both for positive and negative behaviors. An increase of concentration resulting from an emission reduction is linear when changes are proportional.

**Linear component and non-linear deviation term**

The relation between the concentration change and the emission reduction strength (α) around the base case (α=0) can be expressed with the Taylor-Young development. A linearization of the concentration changes in vicinity of the base case is made by only keeping the first-order terms so that:

(A.13) $$P_{\mathrm{all}}^{\alpha }\approx P_{NH3}^{\alpha }+P_{\mathrm{NOx}}^{\alpha }+P_{SO2}^{\alpha }$$

This approximation is valid only for values of α that are close enough to 0. In practice, we estimate in this work that this is the case for α values less than or equal to 10% (α=0.1). For this reduction level, the overall non-linear component, computed as the difference between the sum of the potential impacts of each precursor and the combined potential impact is homogeneous over Europe and, remains everywhere below 4%. (not shown).

The potential impact resulting from the reduction of all precursors can be decomposed into a linear component and a deviation term as follows:

(A.14) $$P_{\mathrm{all}}^{\alpha }=P_{\mathrm{all}}^{\mathrm{lin}}+{\hat{P}}_{\mathrm{tot}}^{\alpha }$$

The linear component is defined as the linearization of the Taylor-Young development (A.13) for an emission reduction percentage of 0.1:

(A.15) $$P_{\mathrm{all}}^{\mathrm{lin}}\approx P_{NH3}^{0.1}+P_{\mathrm{NOx}}^{0.1}+P_{SO2}^{0.1}$$

The overall non-linear deviation is then computed as the difference between the overall potential impact and its linear component:

(A.16) $${\hat{P}}_{\mathrm{tot}}^{\alpha }=P_{\mathrm{all}}^{\alpha }-P_{\mathrm{all}}^{\mathrm{lin}}\approx P_{\mathrm{all}}^{\alpha }-P_{NH3}^{0.1}-P_{\mathrm{NOx}}^{0.1}-P_{SO2}^{0.1}$$

Thanks to Eq. (A.12) the deviation terms can be decomposed into the following components:

$${\hat{P}}_{\mathrm{tot}}^{\alpha }={\hat{P}}_{NH3}^{\alpha }+{\hat{P}}_{\mathrm{NOx}}^{\alpha }+{\hat{P}}_{SO2}^{\alpha }+{\hat{P}}_{NH3-NOx}^{\alpha }+{\hat{P}}_{NH3-SO2}^{\alpha }+{\hat{P}}_{\mathrm{NOx}-SO2}^{\alpha }+{\hat{P}}_{NH3-NOx-SO2}^{\alpha }$$

with ${\hat{P}}_{NH3}^{\alpha }={P_{NH3}^{\alpha }-P}_{NH3}^{0.1},{\hat{P}}_{\mathrm{NOx}}^{\alpha }={P_{\mathrm{NOx}}^{\alpha }-P}_{\mathrm{NOx}}^{0.1},{\hat{P}}_{SO2}^{\alpha }={P_{SO2}^{\alpha }-P}_{SO2}^{0.1}$

**Typical situations**

The analysis of different simulation results reveals three typical situations:

• Situation A (Fig. A.2 top): Reduced emissions lead to reduced concentration with a slope that is steeper for low α values. In such situations, potential impacts are positive and decrease with α. Non-linear deviations are negative and increase with α.
• Situation B (Fig. A.2. center): Reduced emissions lead to reduced concentration with a slope that is steeper for high α values. In such situations, potential impacts are positive and increase with α. Non-linear deviations are positive and increase with α.
• Situation C (Fig. A.2. bottom): Similar to B but reduced emission lead to increased concentrations for low α values. In such situations, potential impacts are negative close to the base case and positive for larger α. Non-linear deviations are positive and increase with α.

## Annex B Classification of the months

A principal component analysis (PCA) and a bottom-up hierarchical cluster analysis are used to classify months according to the chemical regimes (${NH}_{3}$-sensitive, ${NO}_{x}$-sensitive, ${SO}_{2}$-sensitive). This analysis gathered months into three clusters: from November to February the percentages of ${NH}_{3}$-sensitive cells are above their yearly average, from May to September the percentages of ${SO}_{2}$-sensitive cells are above their yearly average, October, March and April the percentages of ${NO}_{x}$-sensitive cells are above their yearly average (Fig. B1, Fig. B2).

## Annex C Additional maps

See Fig. C1, Fig. C2.
